# Complete Genome Sequence of a Foot-and-Mouth Disease Virus of Serotype O Isolated from Gimje, Republic of Korea, in 2016

**DOI:** 10.1128/genomeA.01694-16

**Published:** 2017-03-09

**Authors:** Soyoon Ryoo, Taeseong Kim, Jin Ju Nah, Min Geun Sagong, Sumee Lee, Kwang-Nyeong Lee, Young-Joon Ko, Jong-Hyeon Park, Myoung-Heon Lee, Sung-Hwan Wee, Dongseob Tark, Bok Kyung Ku

**Affiliations:** Foot-and-Mouth Disease Division, Animal and Plant Quarantine Agency, Gimcheon, Gyeongsangbuk-do, Republic of Korea

## Abstract

The complete genome sequence of a foot-and-mouth disease (FMD) serotype O virus isolated from Gimje, Republic of Korea, is reported here.

## GENOME ANNOUNCEMENT

Foot-and-mouth disease virus (FMDV) belongs to the genus *Aphthovirus* in the family *Picornaviridae* and causes a highly contagious vesicular disease in cloven-hoofed animal species. FMDV is divided into seven immunologically distinct serotypes, A, O, C, Asia 1, and South African Territories (SATs) 1, 2, and 3. FMDV type O is the pandemic serotype and is grouped into eight topotypes: Cathay, Middle East-South Asia (ME-SA), Southeast Asia (SEA), Europe-South America (Euro-SA), Indonesia-1 and -2 (ISA-1 and -2, respectively), East Africa (EA), and West Africa (WA), based on 15% nucleotide differences (1). On 11 January 2016, FMD was definitively diagnosed for the first time in Gimje, Jeollabuk-do, Republic of Korea. The FMD viral isolates were of serotype O, indicating that they were related to the viral strains of the SEA topotype that are circulating in East Asian countries (2).

Here, we report the complete genome sequence of an FMDV serotype O strain (O/GJ/SKR/2016) that was isolated on 11 January 2016 from vesicular fluid collected from an infected pig from Gimje. Viral RNA was extracted from the cell culture supernatant of the BHK-21 cell line, and cDNA was synthesized using random and oligo(dT) primers with SuperScript III reverse transcriptase (Thermo Fisher Scientific). We designed pairs of primers to produce 20 overlapping amplicons spanning the entire viral genome based on the sequence of the O/SEA/Mya-98 lineage (3). Sequence analyses were performed using SeqMan Pro version 12 (DNAStar Lasergene, USA).

The complete genome of strain O/GJ/SKR/2016 was 8,132 nucleotides (nt) in length, including a 1,011-nt 5′ untranslated region (5′ UTR) with an 18-nt poly(C) tract and a 122-nt 3′ UTR with a ≥29-nt poly(A) tail. A single open reading frame (ORF) of 6,999 nt was predicted to encode 2,333 amino acids containing four structural and 10 nonstructural proteins. The most closely related publicly available complete genome sequence to O/GJ/SKR/2016 was isolated in 2014 from Jincheon, Republic of Korea (O/SKR/JC/2014, GenBank accession no. KX162590.1), with which it shared 98.8% nucleotide and 98.7% amino acid identity. As a result of analysis of the VP1 gene, the nucleotide and amino acid of O/GJ/SKR/2016 showed 99.06% identity with O/SKR/JC/2014.

### Accession number(s).

The complete genomic sequence of O/GJ/SKR/2016 has been deposited in GenBank under accession no. KY086465.
